# Study of a constrained finite element elbow prosthesis: the influence of the implant placement

**DOI:** 10.1186/s10195-023-00690-x

**Published:** 2023-04-13

**Authors:** Lorenzo Nalbone, Francesco Monac, Luca Nalbone, Tommaso Ingrassia, Vito Ricotta, Vincenzo Nigrelli, Massimo Ferruzza, Luigi Tarallo, Giuseppe Porcellini, Lawrence Camarda

**Affiliations:** 1 Department of Orthopedic and Traumatology (DICHIRONS), Università degli Studi di Palermo, Via del Vespro, 90100 Palermo, Italy; 2 Department of Veterinary Science, Università degli Studi di Messina, Messina, Italy; 3 Department of Engineering, Università degli Studi di Palermo, Palermo, Italy; 4 Department of Orthopedic and Traumatology, Università degli Studi di Modena, Modena, Italy

**Keywords:** Totel elbow arthroplasty, Biomechanics, Elbow replacement, Prosthetic posizioning, Elbow finite element

## Abstract

**Background:**

The functional results of total elbow arthroplasty (TEA) are controversial and the medium- to long-term revision rates are relatively high. The aim of the present study was to analyze the stresses of TEA in its classic configuration, identify the areas of greatest stress in the prosthesis–bone–cement interface, and evaluate the most wearing working conditions.

**Materials and methods:**

By means of a reverse engineering process and using a 3D laser scanner, CAD (computer-aided drafting) models of a constrained elbow prosthesis were acquired. These CAD models were developed and their elastic properties, resistance, and stresses were studied through finite element analysis (finite element method—FEM). The obtained 3D elbow-prosthesis model was then evaluated in cyclic flexion–extension movements (> 10 million cycles). We highlighted the configuration of the angle at which the highest stresses and the areas most at risk of implant mobilization develop. Finally, we performed a quantitative study of the stress state after varying the positioning of the stem of the ulnar component in the sagittal plane by ± 3°.

**Results:**

The greatest von Mises stress state in the bone component for the 90° working configuration was 3.1635 MPa, which occurred in the most proximal portion of the humeral blade and in the proximal middle third of the shaft. At the ulnar level, peaks of 4.1763 MPa were recorded at the proximal coronoid/metaepiphysis level. The minimum elastic resistance and therefore the greatest stress states were recorded in the bone region at the apex of the ulnar stem (0.001967 MPa). The results of the analysis for the working configurations at 0° and 145° showed significant reductions in the stress states for both prosthetic components; similarly, varying the positioning of the ulnar component at 90° (− 3° in the sagittal plane, 0° in the frontal plane) resulted in better working conditions with a greater resulting developed force and a lower stress peak in the ulnar cement.

**Conclusion:**

The areas of greatest stress occur in specific regions of the ulnar and humeral components at the bone–cement–prosthesis interface. The heaviest configuration in terms of stresses was when the elbow was flexed at 90°. Variations in the positioning in the sagittal plane can mechanically affect the movement, possibly resulting in longer survival of the implant.

*Level of evidence*: 5

## Introduction

Total elbow prosthesis (TEA—total elbow arthroplasty) is an orthopedic surgical technique that involves the total replacement of the elbow joint. First performed in 1925 by Robineau [1], it was initially proposed for the treatment of rheumatoid arthritis. Since then, TEA has also found increasing use for other pathologies, such as complex acute fractures in patients with low functional demands or in the treatment of post-traumatic arthritis. The goal of this surgery is to restore the main characteristics of a normal elbow, in the absence of pain and instability, with good mobility [2]. Currently, TEAs are essentially divided into two types: linked and unlinked. The difference lies in the level of "constraint," i.e., the ability of the prosthesis to resist dislocation [3]. The unlinked variants do not have any element of constriction between the ulnar and humeral components, so they do not have intrinsic resistance to stress in a dislocative sense. Therefore, perfect integrity of the surrounding soft tissues is required to stabilize the neo-articulation. Linked-type TEAs, on the other hand, have a constraint in the hinge that unites the two prosthetic semi-components which limits their rotation along the axis perpendicular to the rotation axis of the hinge. In reality, the most recent models, as well as those currently most used, have a certain secondary mobility in internal–external rotation and in the varus–valgus one (semi-constrained linked models) [3]. This is because constrained prostheses, over time, revealed high loosening rates due to the high stress at the prosthesis–bone–cement interface, which leads to excessive wear of the components. Moreover, high constriction does not reproduce the original anatomy of the elbow.

Although there has been a certain increase in the number of TEA operations, this number remains far below that of prosthetic replacements of joints such as the hip, knee, and shoulder, which have a shorter survival and a higher rate of reoperation. Basically, in elbow prosthetic replacement, there is a higher rate of revision compared to other more prosthetic and less frequently revised joint districts. The data reported in the literature show that most cases of elbow prosthesis failure are due to aseptic loosening phenomena (18–48%) [3–5], polyethylene wear (11–20%) [3], and, more generally, structural failure of the components [6]. All events are probably due to the overuse of the prosthetic limb.

To date, there is little literature available on TEA positioning, on the biomechanical functioning of a TEA, and on how possible modifications of the implants, the mounting configuration, and the surgical technique can improve the functionality of a prosthetic elbow and consequently the long-term survival.

The aim of our work was to study the tension stresses, the different geometric configurations, and the positioning of the prosthetic components. This was performed to identify the optimal prosthetic configuration and positioning in order to evaluate the reduction in stress conditions during flexion–extension movement in the aforementioned bone–prosthesis–cement interface.

## Materials and methods

**Fig. 1:**
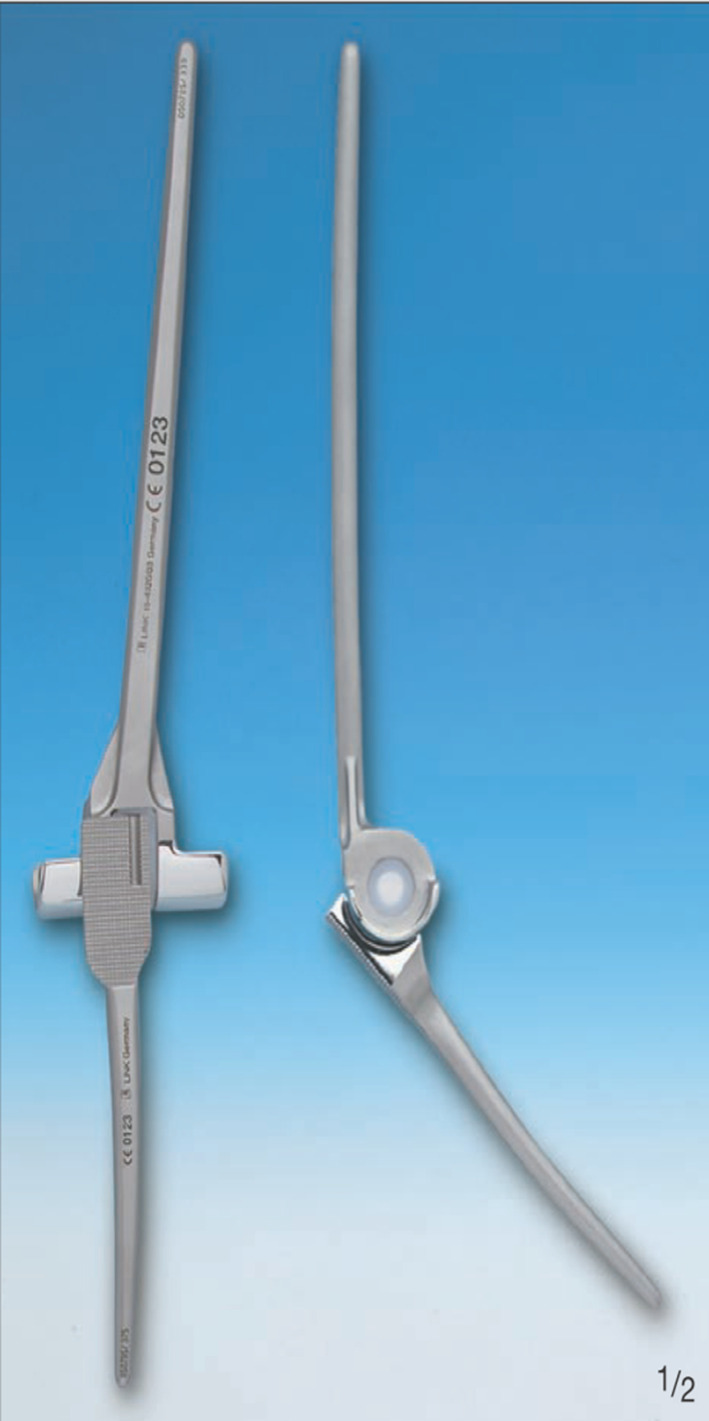
LINK^®^ Endo-Model^®^ elbow prosthesis

**Fig. 2:**
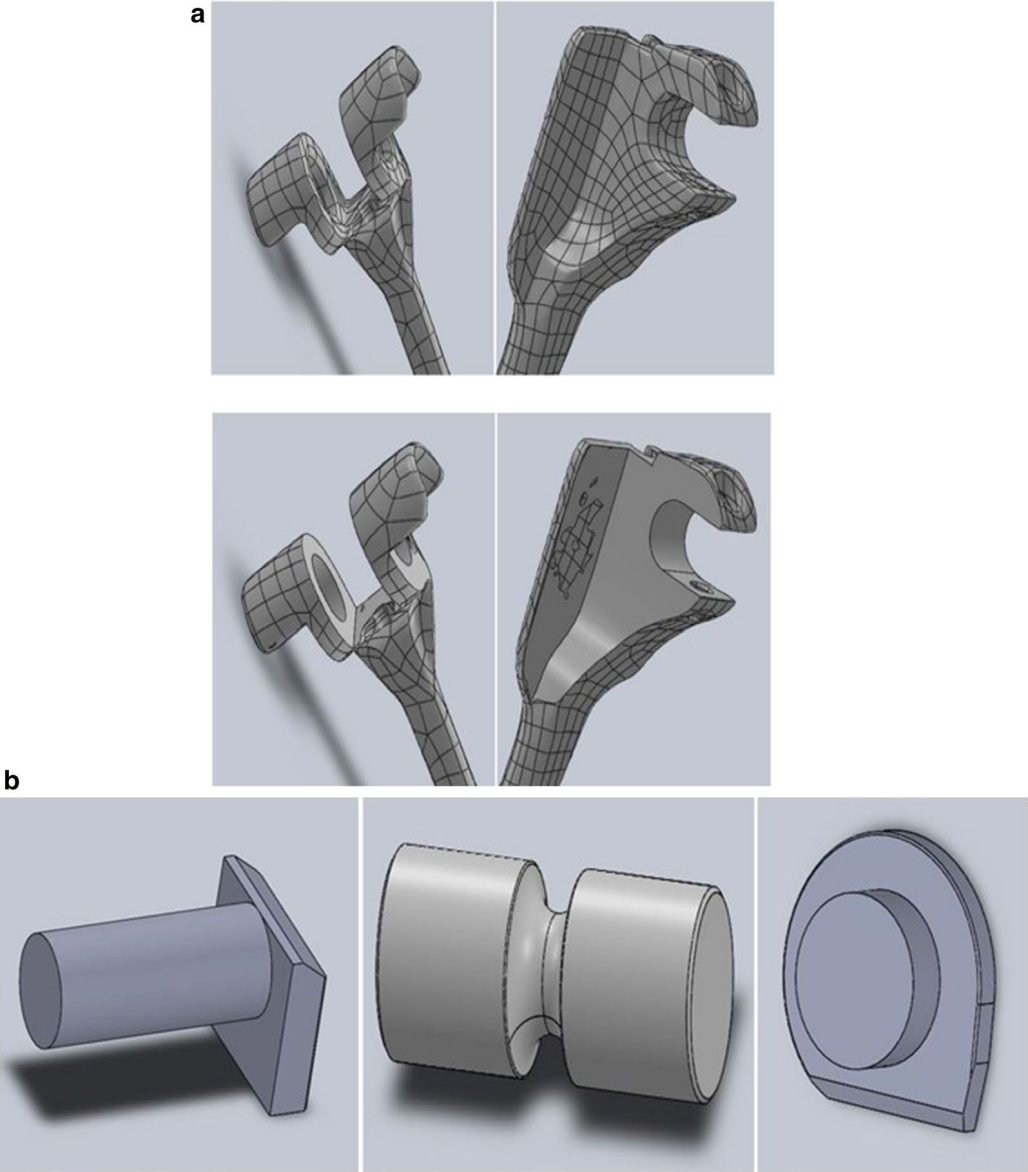
**a** CAD model of the ulnar and humeral components. **b** CAD model of the polyethylene insert

**Fig. 3:**
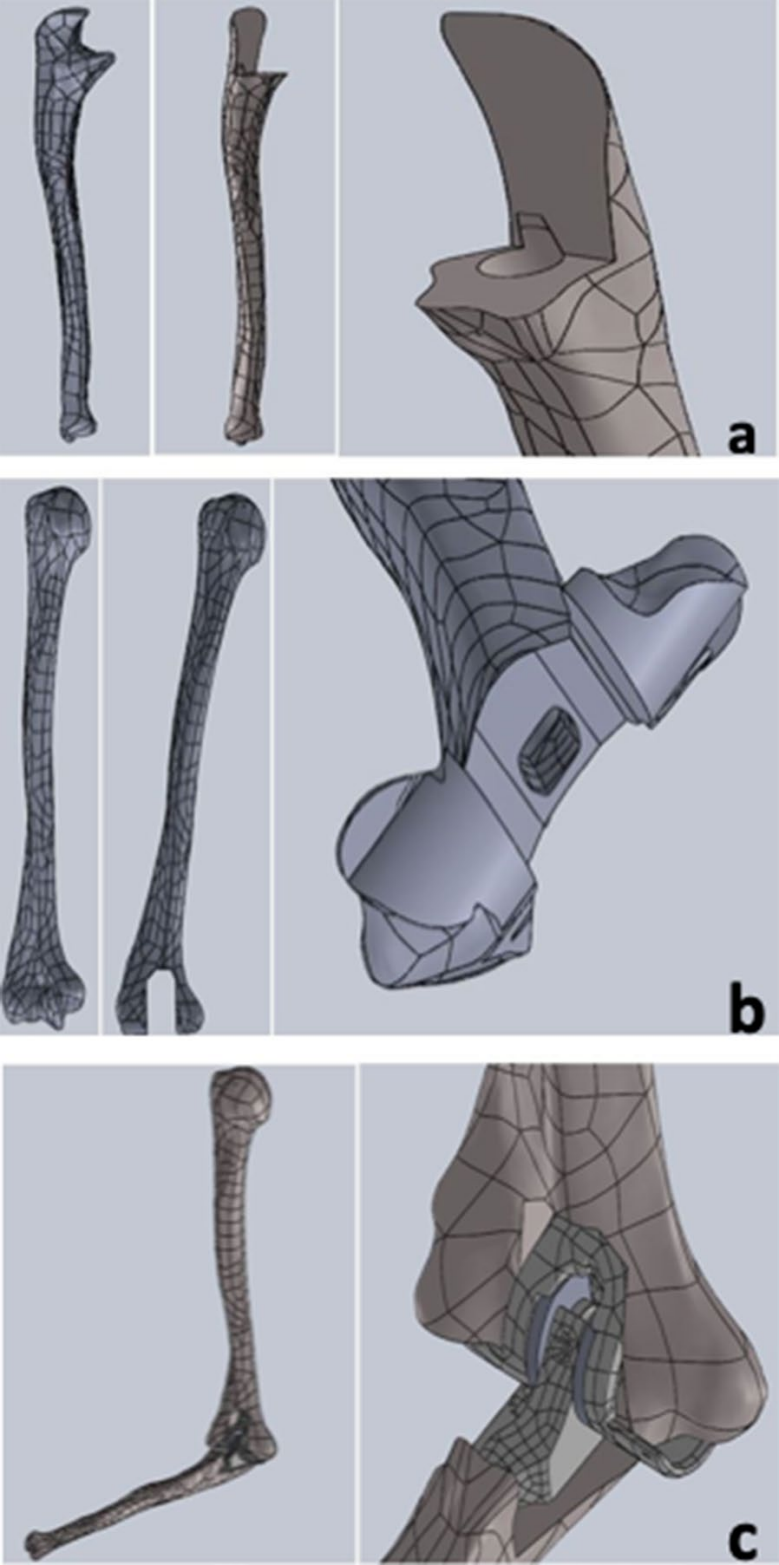
CAD model of the ulna (**a**), humerus (**b**), and post-implant elbow prosthesis (**c**)

**Fig. 4:**
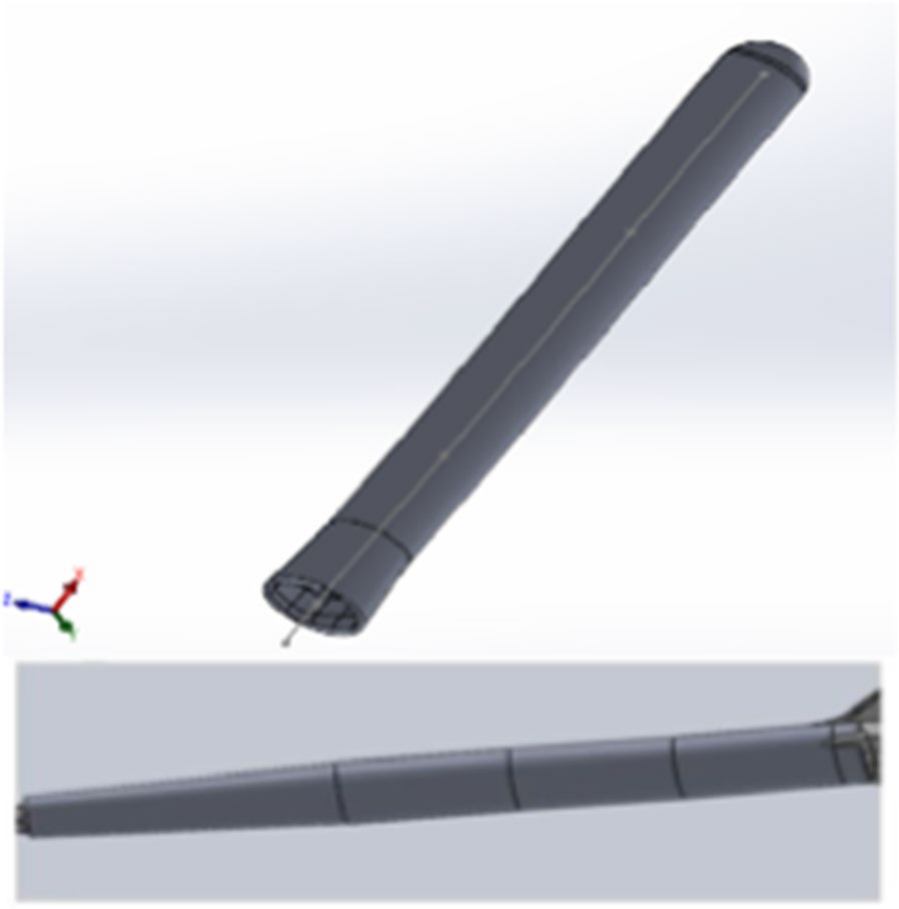
Lining of the humeral and ulnar canals with cement film

To study the behavior of an implanted elbow prosthesis and analyze its response at the bone–cement–prosthesis interface, a linked model was considered, in particular a LINK Endo-Model^®^, the third generation of the St. George prosthesis, without a radial capital component (Fig. 1). The prosthesis, made from chromium-cobalt alloy, was developed in a 3D model with the aid of a 3D scanner, through which—with the projection of a cloud of points onto the components—it was possible to generate the first CAD (computer-aided drafting) model. The prosthesis was subsequently manipulated with the SolidWorks software (SW) to make it as close to reality as possible (Fig. 2a, b). Bone components were obtained from the BodyParts3D Project online database, which has a set of anatomically reliable components of the human body. These were modified, by SW, to allow correct assembly with the prosthetic components. The modification work was carried out in accordance with the literature to best reflect TEA implantation (Fig. 3). The modeling of the cement film was also carried out with SW through a process of Boolean subtraction, starting from the hole in the bone and the shape of the prosthesis (Fig. 4) and assuming a cement thickness of 1 mm in the humeral canal and an average cement thickness of 0.5 mm in the ulnar canal, given the varying diameter of the bone along its length.

**Fig. 5:**
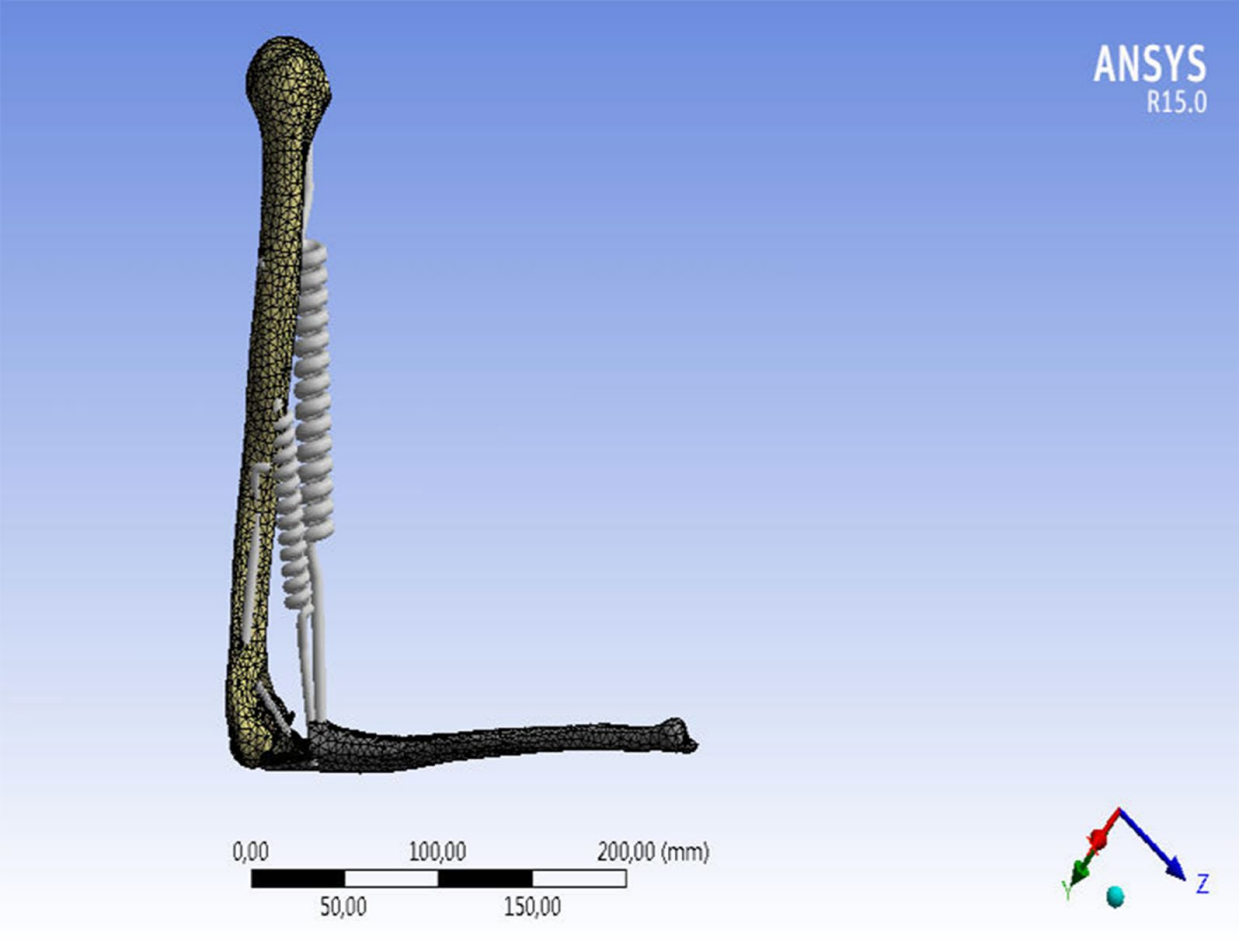
Ansys Student musculoskeletal biomechanical representation of the elbow

After the modeling, we proceeded with the finite element analysis using Ansys Student software. The preprocessor setting was carried out as follows:

- Meshing was carried out with the Hex20 type element, with different average element sizes used for each body
- A fixed type constraint was applied in the humerus head
- A force in the proximal ulna that was a function of the angle of opening of the prosthesis was applied, in accordance with the literature [7–11]
- Springs anchored in the fixing points of the various muscles and designed to emulate their behavior were applied (Fig. 5).

**Fig. 6:**
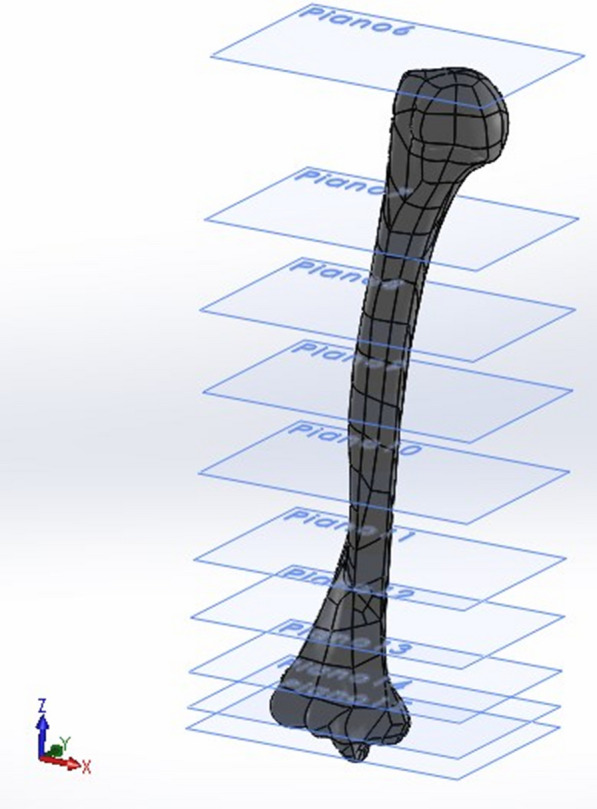
Humeral model secant planes

This procedure was carried out for three different working configurations of the prosthesis (0°, 90°, and 145°). After analyzing each of them, the improvement action was subsequently undertaken in order to find a mounting configuration that would minimize the tension at the bone–prosthesis–cement interface for a static load of 2 kg, with the recommended working conditions simulated for repetitive flexion–extension efforts (> 10 million cycles) in a prosthetic patient. The analysis began with the generation of a number of "satisfactory" planes which cut the models at different heights along their lengths (Fig. 6). Each of these planes intersecting the model gave rise to a cross section whose center-of-gravity coordinates were collected. Using the MatLab calculation software, a 3D linear regression was then carried out with the aforementioned points to obtain the coordinates of the points of the line that best represented these centers of gravity. In this way, the longitudinal axes of the humerus, ulna, and the related prosthetic axes were obtained.

**Fig. 7:**
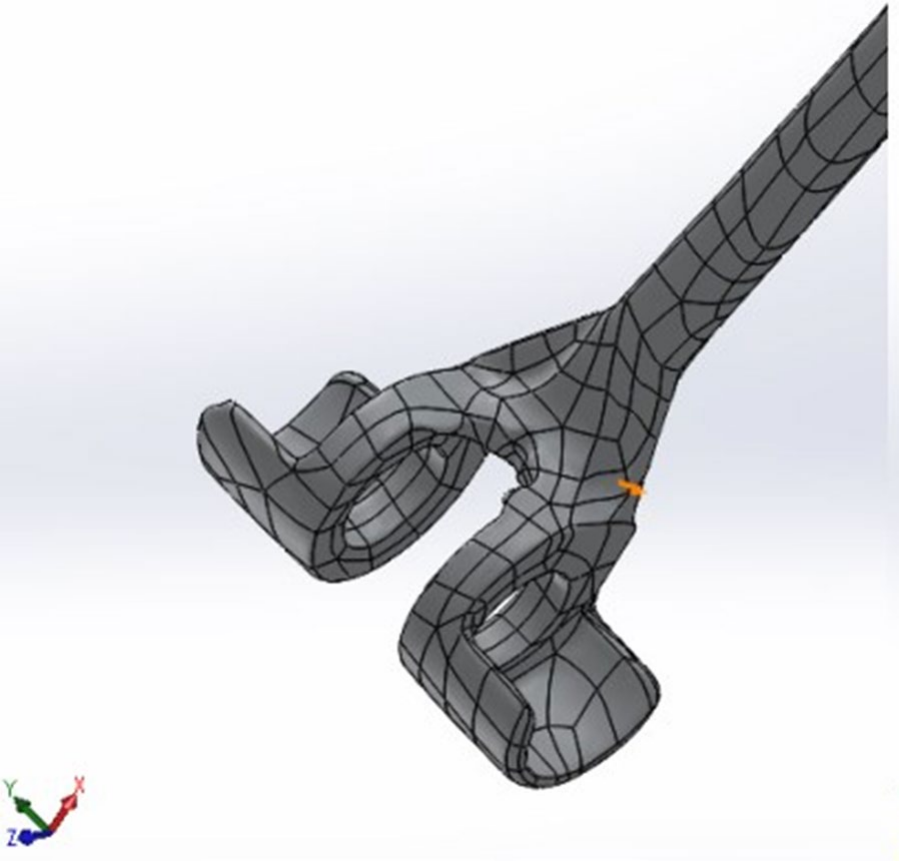
Cleaning the surfaces

**Fig. 8:**
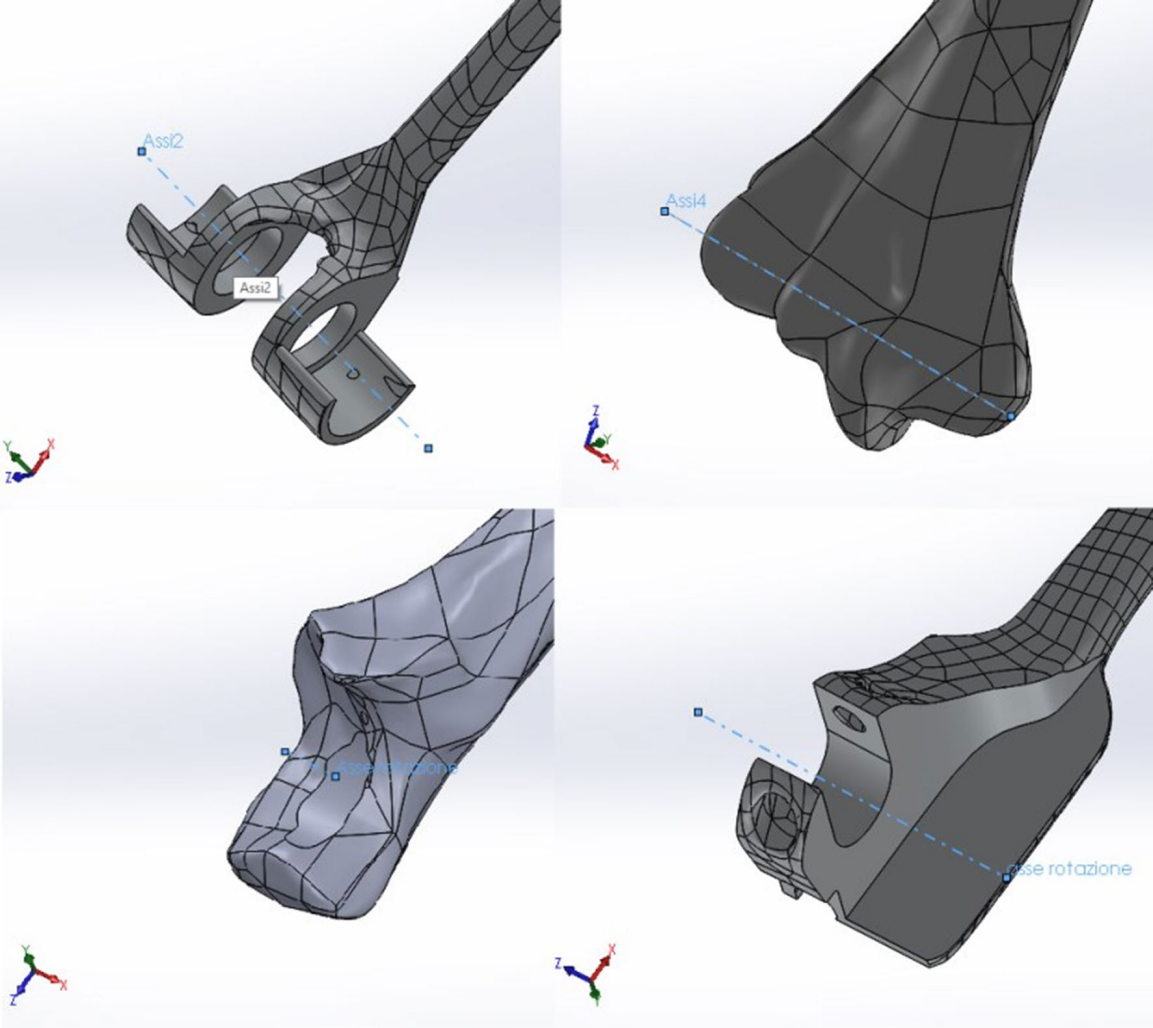
Humero-ulnar rotation axes

**Fig. 9:**
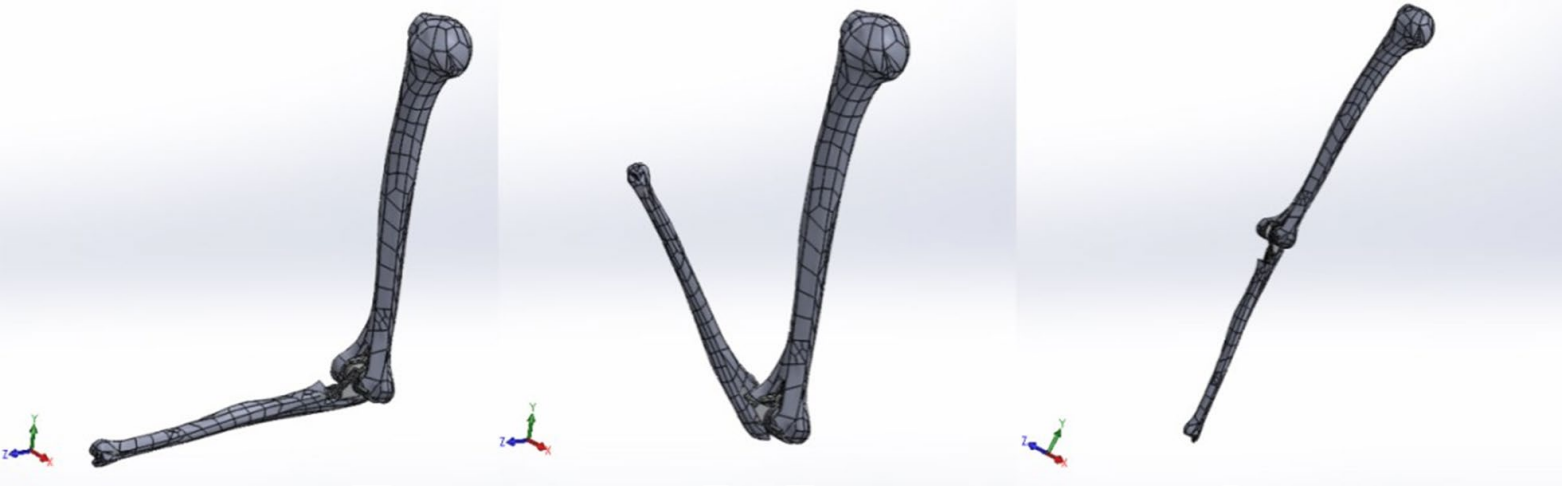
Bone-prosthesis models for different angle configurations (90°, 145°, and 0°)

In order to reduce the computational load of the FEM analysis, it was decided to clean the surfaces of the prosthesis models—having been obtained using a 3D scanner, they each had a mesh that made the surface rather irregular. It was considered acceptable to bore the holes and flatten the surfaces by removing material: the objective of the study was, in fact, to analyze the stress state in the cement and not the response of the prosthesis (Fig. 7). After that, it was possible to proceed with the final bone–prosthesis assembly (Fig. 8) in the three configurations proposed in the study at 90°, 145°, and 0° (Fig. 9). At this point, the intrinsic characteristics of the bone, the materials used to construct the prosthetic components, and the cement were considered as boundary conditions in terms of the modulus of elasticity (*E*), Poisson's ratio, or transversal contraction coefficient (*ν*).

**Fig. 10:**
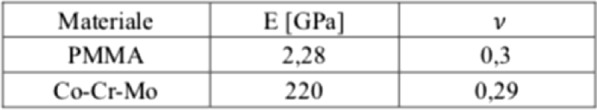
Material properties

**Fig. 11:**
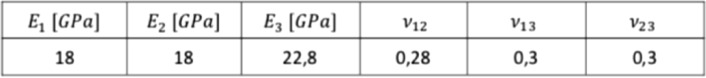
Elastic and resistance properties of cortical bone (*E*_1_ and* ν*_12_), trabecular bone (*E*_2_ and* ν*_13_), medullary bone (*E*_3_ and* ν*_23_) [9]

The prosthesis is made of a chromium-cobalt-molybdenum (Co-Cr-Mo) alloy, while the cement is a thermoplastic polymer of polymethylmethacrylate (PMMA). In particular, the characteristics of PMMA were obtained from the data provided by Completo et al. [7], the characteristics of the metals in the prosthesis (Co-Cr-Mo aluminum alloy) were obtained from the makeitfrom.com website (Fig. 10), and the characteristics of the cortical, trabecular, and medullary bone (Fig. 11) were obtained from a study carried out by Isaza et al. [8].

**Fig. 12:**
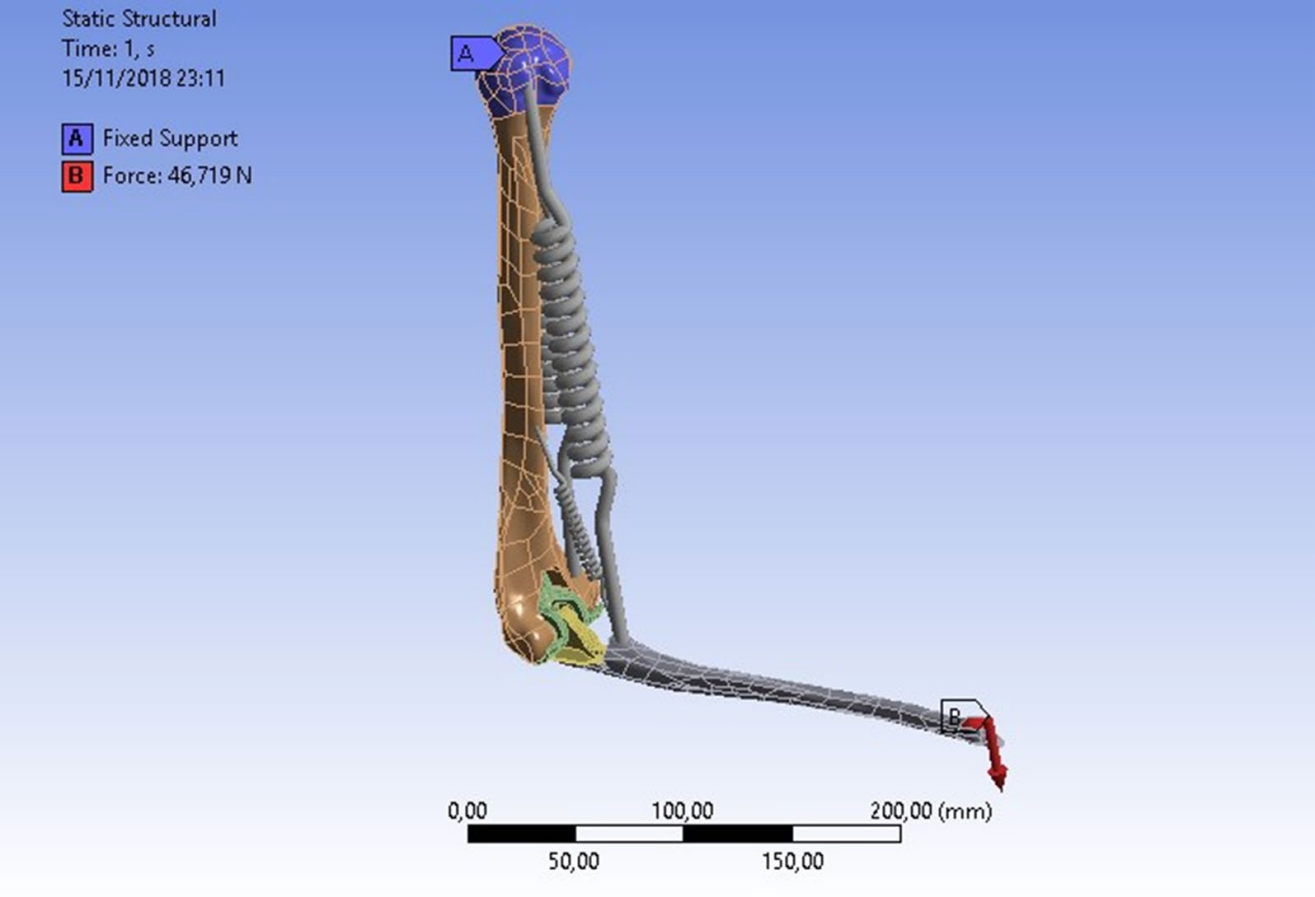
Final 90° configuration

As for the loads, it was decided that a force would be applied to the distal ulna which reflects the optimal conditions for each of the chosen angle configurations between arm and forearm, and the total moment arm that the muscles exert was then determined; from this bending moment, the resulting force on the extremity of the ulna was subsequently determined. The muscles were simulated as springs whose elastic coefficient* k* is large enough for them to avoid being stretched. The idea was to test the model when the muscles exert their maximum force, which consequently corresponds to the maximum force that they would be able to withstand. We therefore arrived at the final 90° configuration (Fig. 12) in which the shoulder is blocked, the muscles have a fixed length (isometric contraction), and the force on the wrist is equal to the moment for the given angle divided by the length of the ulna. The next step was to set the contact conditions between all elements of the model. In the cement–prosthesis and cement–bone interfaces, a bonded type relationship was set, which causes the bodies to be “glued” to each other. To avoid increasing the computational load too much, it was decided to replace the polyethylene parts (which would make it possible to rotate the arm and forearm) with a revolute constraint between the two prosthetic components. On the other hand, frictionless relationships were established between prosthetic elements and related bones. All this was proposed for the three angle configurations between arm and forearm that were taken into consideration (0°, 90°, and 145°), with the aim being to identify the most onerous configuration and subsequently to apply, by trial and error, modifications in the assembly. In a final analysis, in fact, changes were made in the assembly of the ulnar prosthetic component; in particular, the angle (3°) between the prosthetic axis and the bone axis or in the sagittal or frontal plane was varied. The four resulting configurations (A, B, C, D) are shown in Table 1.

**Table 1:** 4 resulting configurations (A - B - C -D)

	Sagittal (°)	Front (°)
A	− 3°	0°
B	+ 3°	0°
C	0°	− 3°
D	0°	+ 3°

## Results

**Fig. 13:**
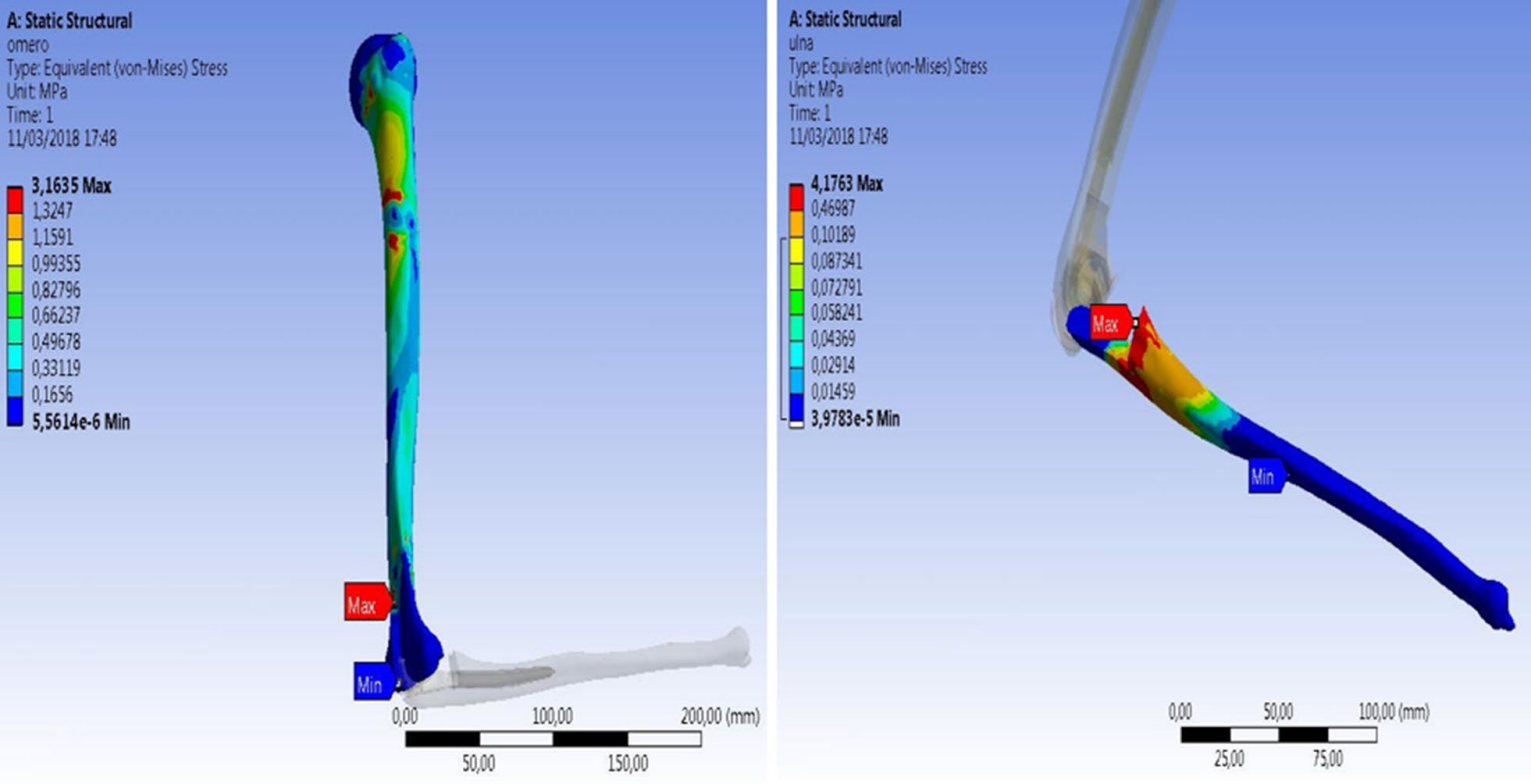
Maximum and minimum humeral/ulnar strain: 90° configuration

The first analysis to be presented is that relating to the 90° configuration between arm and forearm. First of all, the stress states at the bone level were recorded, measuring them in terms of the von Mises criterion or criterion of maximum distortion at both the ulna and the humerus (Fig. 13). In particular, as can be seen from the image, the major von Mises stress states (3.1635 MPa) at the humeral level were recorded in the most proximal portion of the humeral blade and in the proximal middle third of the shaft. At the ulnar level, on the other hand, the major stress states (4.1763 MPa) were highlighted at the level of the coronoid.

**Fig. 14:**
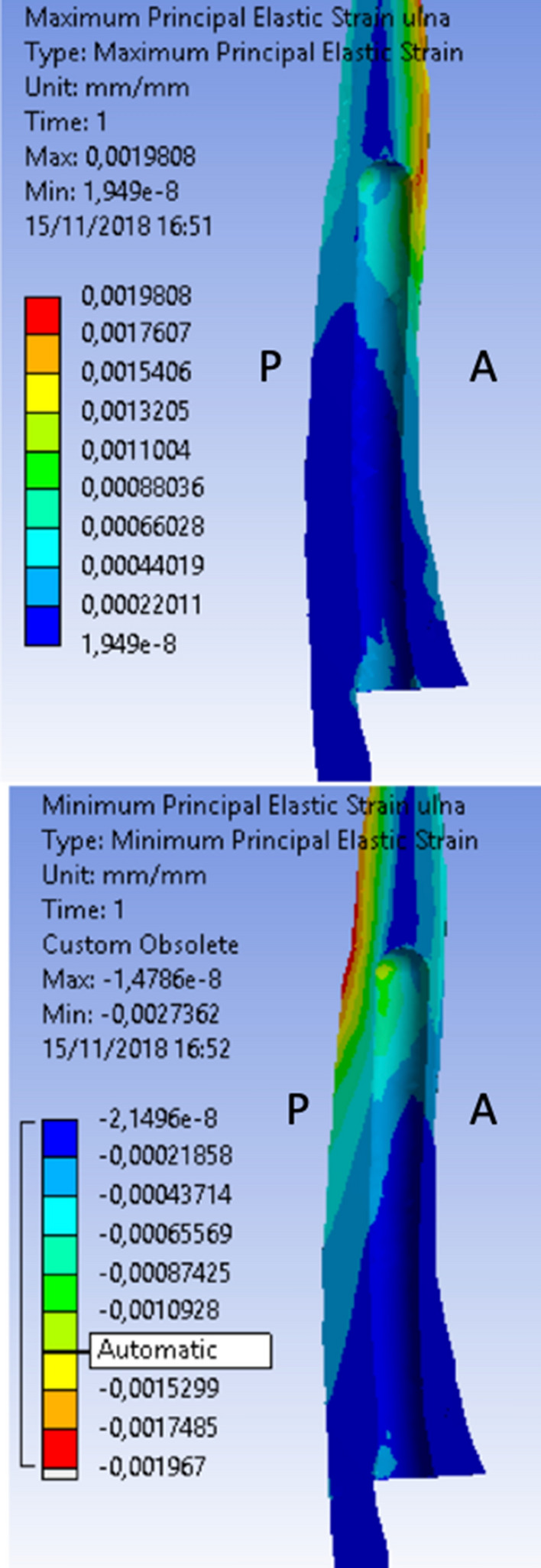
Maximum and minimum ulnar component strain: 90° configuration

The results obtained for the maximum and minimum post-implantation bone strain of the ulnar prosthetic component are those shown in Fig. 14. In this case, the minimum elastic resistance and therefore the greatest stress states were recorded in the bone region at the apex of the ulnar stem and in particular in the posterior cortex (P) of the ulnar shaft (0.001967 MPa). In order to reduce the computational load and the possible combinations, it was decided to proceed with the analyses by taking into consideration only the forearm, not the humeral part, and by trying to reproduce the boundary conditions in such a way that the analyses are minimally compromised by the lack of the humeral part.

**Fig. 15:**
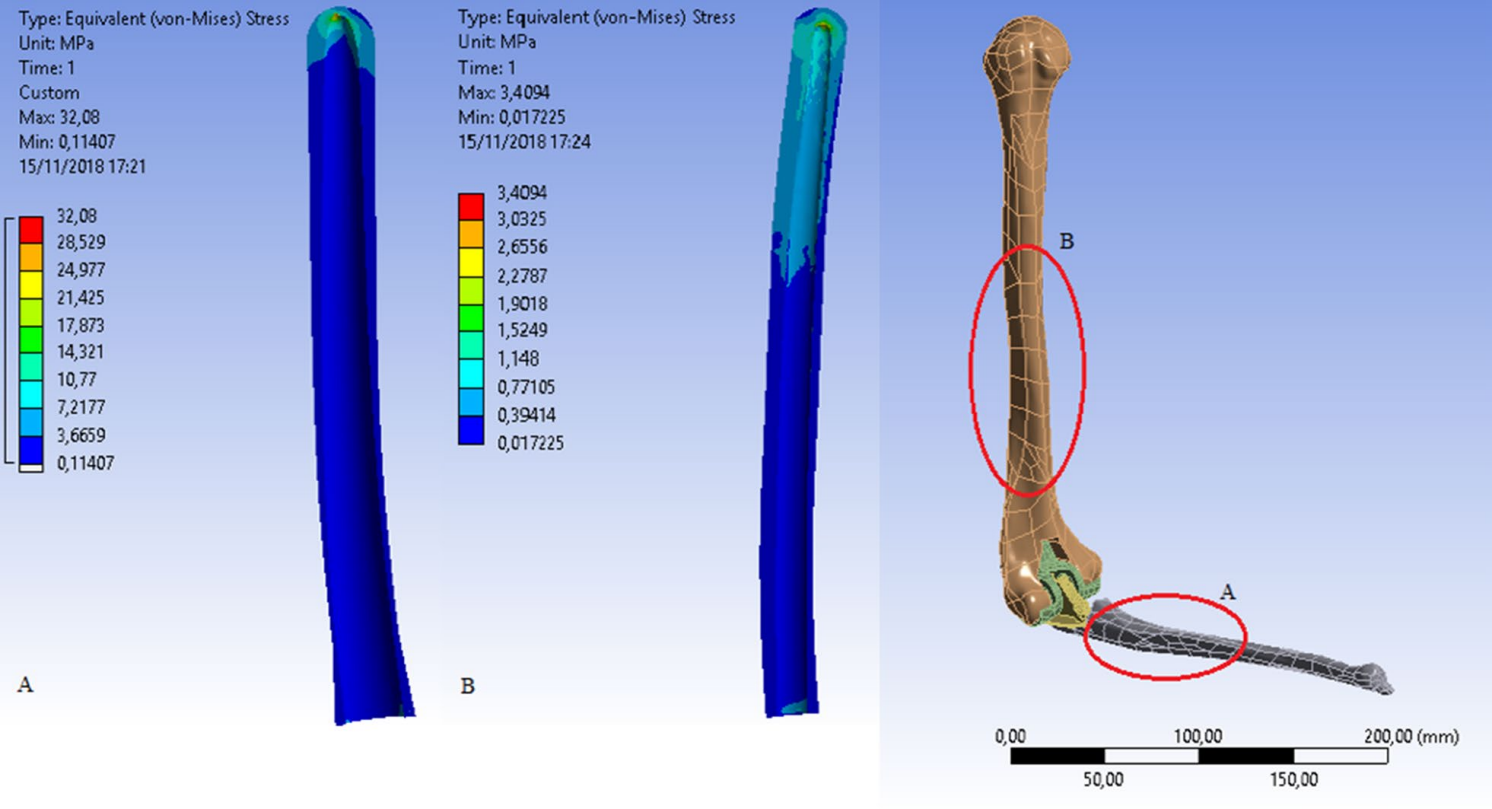
Ulnar (**A**) and humeral (**B**) cement tension states: 90° configuration

**Fig. 16:**
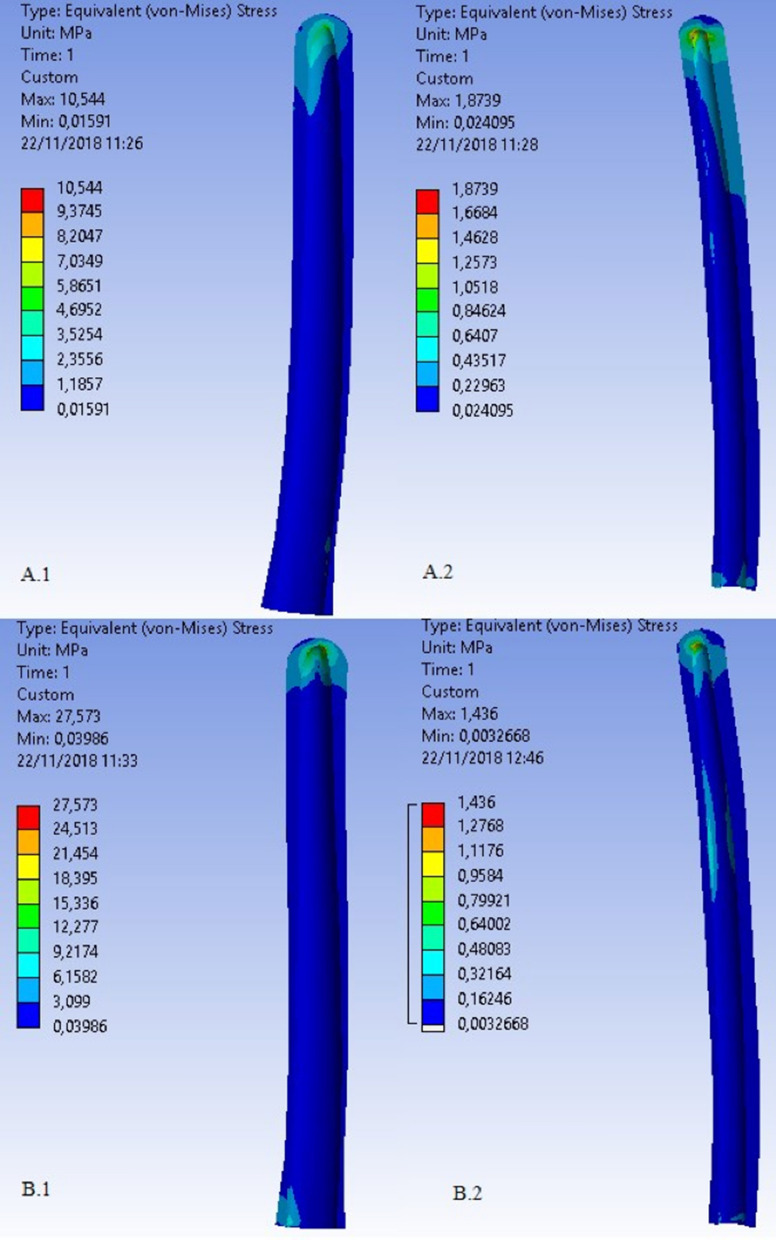
von Mises tensions.* Top*: 0° configuration (**A.1** ulnar cement; **A.2** humeral cement). *Bottom*: 145° configuration (**B.1** ulnar cement; **B.2** humeral cement)

The von Mises stress states were therefore also recorded at the level of the concrete lining layer. The results obtained are illustrated in Fig. 15. Specifically, there was a higher von Mises tension in the veneering cement at the apex of the ulnar stem (A max = 32.08 MPa) and in the veneering cement at the apex of the humeral stem (B max = 3.4094 MPa). To determine which of the angles of the elbow involves the heaviest working conditions for the bone-–cement interface, the configurations of the model in which the angle between arm and forearm was 0° or 145° were taken into consideration. The data obtained are shown in Fig. 16.

The results show peaks in the von Mises stresses of 10.544 MPa and 27.573 MPa for the ulnar components in the 0° and 145° configurations, respectively. The maximum values recorded were, on the contrary, 1.8739 MPa and 1.436 MPa for the humeral components in the same configurations (Table 2). The data were obtained in terms of stress states in the bone component alone (4.1763 MPa), in the post-implantation bone (0.001967 MPa), and in the coating cement layer (32.08 MPa). These show that maximum tension is localized in the ulna near the apex of the prosthetic stem. Of all the configurations chosen, the one that leads to the worst load condition is that at 90°, and, in particular, the maximum peak is located in the ulnar component. For this reason, we decided to focus our research on a better mounting configuration in the 90° model and exclusively on the ulnar component. Contrary to configurations B, C, and D shown in Table 1, which keep the stress state roughly unchanged, configuration A shows a decrease in the maximum peak, which falls from about 29 MPa to about 22 MPa (Table 3). Therefore, two models were studied at 90°: (1) with the standard configuration (STD) of the ulnar component and (2) with a mounting configuration of − 3° in the sagittal plane (configuration A in Table 1). The results obtained are summarized in Table 4.

**Table 2:** Peak in the von Mises Stress for different ulnar components configurations

	Peak von Mises stress	
	Ulnar cement (MPa)	Humeral cement (MPa)
0°	10.544	1.8739
90°	32.08	3.4094
145°	27.573	1.436

**Table 3:** Peak tension in the ulnar side on different configurations

	Resulting force (N)	Peak tension in the ulnar cement (MPa)
A: − 3°/0°	261.53	22.387
B: + 3°/0°	254.55	29.395
C: 0°/− 3°	254.27	33.381
D: 0°/ + 3°	254.47	34.152

**Table 4:** Results of models studied at 90° of elbow flexion  1) standard configuration.  2 ) ulnar component with −3° in the sagittal plane

	Brachial (N)	Brachial biceps (N)	Elbow strength (N)	Peak von Mises stress in ulnar cement (MPa)
90° standard configuration	106.98	27.515	250.59	32.08
90° configuration A (− 3°/0°)	104.2	31.9	257.71	22.387

## Discussion

The main result in the present study is that during flexion–extension movement with a load of 2 kg applied distally to the elbow, as occurs during minimal daily activity, a high stress on the bone and cement is generated. The greatest concentration of these stresses in the von Mises equivalent is localized in the distal part of the humeral component and in the most proximal part of the ulnar component. These are the areas where a high concentration of stress could cause implant failure if the applied force is prolonged [18]. Further, the displacement of the axis of the ulnar stem by −3° in the sagittal plane results in less stress, proving that variations in the positioning of the components with respect to the anatomical axis can lead to a potential improvement in biomechanics and therefore a longer predicted survival of the prosthesis.

There are various types of TEA, which, based on the connection between the humeral and ulnar components, can be categorized into constrained, semi-constrained, and unconstrained. Currently, most systems are semi-constrained. Therefore, they present a certain degree of laxity at the junction of the components. In the present study, the authors decided to use a constrained system in order to reduce the computational load of the FEM analysis and to evaluate the results as a whole rather than to consider the two semi-prostheses as separate entities. A choice was also made to cement the components, since, as stated by Fevang et al. [9], cemented prostheses have a higher survival rate than non-cemented prostheses.

The results of TEA have improved over the last few decades, and this can be attributed primarily to better biomechanical knowledge of joints and implants. TEA, moreover, is a technically demanding orthopedic procedure in which the precision of the restoration of the center of rotation of the implant as well as the correct positioning of the components are associated with better functional results, fewer complications, and therefore longer survival. However, high revision rates are reported, probably due to aseptic loosening from incorrect alignment between the axis of the constraint and the anatomical axis of rotation. In fact, due to aseptic loosening, rates of implants are reported to be between 47 and 77% with various types of prostheses [7]. Brownhill and Shuind have in fact shown in vitro that bad positioning of the ulnar or humeral component modifies the kinematics of the artificial joint and can determine its “loosening” [10, 11]. Few articles describe the effects of prosthetic implant placement on the stresses on the cement and bone for both the ulna and the humerus. Even less is known about the distribution of stress in this joint. Ericson et al. observed that the movement model of a TEA is, however, much less constrained than a normal elbow and this is more evident in unconstrained implants, with regard to the integrity of muscles and ligaments [12]. The humeral stem is typically valgus to fit the medullary canal; however, in some models, it is perpendicular to the hinge. Therefore, in terms of size and morphology, it must be well cemented, since the humeral canal tends to widen distally and has a thin cortex. On the other hand, the ulnar stem adapts well to the medullary canal of the ulna, which also has a thicker cortex. In 1986, Harry E. Figgie [13] described how small changes in the alignment of the implant with respect to the anatomical structure of the elbow have great effects on the functional results due to the high forces that develop in the joint during flexion and extension.

In a retrospective study on 25 patients operated on for TEA for rheumatoid arthritis or elbow fracture, Lenoir [14] evaluated the clinical outcomes, pain, and functionality of the prosthetic elbow in correlation with the correct positioning of the prosthetic components. Using computed tomography, the anterior offset, lateral offset, valgus, height, and rotation for the ulnar and humeral parts were examined. These indices provided a quantitative assessment of how position errors for the two components had additive or, conversely, counterbalanced effects on each other. The discrepancy between the humeral and ulnar lateral offsets was significantly associated with pain intensity and the Mayo Elbow Performance Score (MEPS); an anterior position of the ulna relative to the humerus was associated with reduced extension force and poorer outcomes for all functional parameters.

**Fig. 17:**
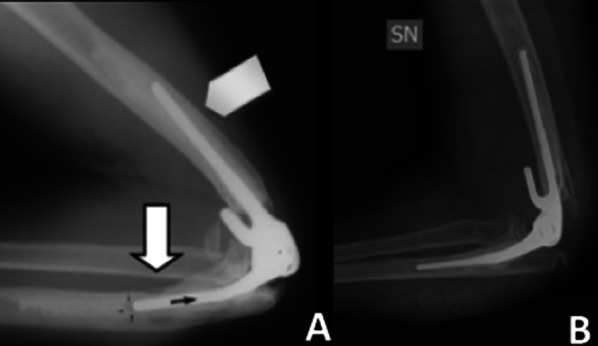
Incorrect positioning (**A**) vs. correct positioning (**B**) of the prosthesis/bone axis

Even in the absence of implant loosening, positioning errors still seem to negatively affect the functional results, probably exerting inappropriate stress on the soft tissues. In Lenoir's study, it was also argued that neither the valgus index nor the rotation index is associated with clinical outcomes [14]. Ultimately, a slight posterior offset of the humeral component and a slight anterior offset of the ulnar component is recommended. In the frontal plane, the implants should be aligned with the native anatomical axes, as shown in Fig. 17.

The main limitation of the present study is represented by the use of simplified mathematical models that may not be fully comparable to complex anatomical situations in vivo; also, it is not possible to relate the study to long-term implant survival. Further, our evaluation takes into account the most common stresses in flexion–extension and not in pronation–supination movements, so it is not possible to evaluate torsional stresses. However, it can be considered that recommending modest activity in daily life may reduce the risk of possible mobilization of the prosthetic components [15–18].

## Conclusions

The areas of greatest stress occur in specific regions of the ulnar and humeral components at the bone–cement–prosthesis interface. The heaviest configuration in terms of stresses is the one with an elbow flexion angle of 90°, with the stresses mainly concentrated in the ulnar component. Variations in positioning in the sagittal plane can mechanically affect the movement, possibly resulting in longer survival of the implant. For this reason, a slight posterior offset of the humeral component and a slight anterior offset of the ulnar component are recommended.

## Data Availability

The dataset analyzed in this study is available from the corresponding author on request.
